# Drop-out and ineffective treatment in youth with severe and enduring mental health problems: a systematic review

**DOI:** 10.1007/s00787-023-02182-z

**Published:** 2023-03-07

**Authors:** R. de Soet, R. R. J. M. Vermeiren, C. H. Bansema, H. van Ewijk, L. Nijland, L. A. Nooteboom

**Affiliations:** 1 LUMC Curium–Department of Child and Adolescent Psychiatry, Leiden University Medical Center, Post Box 15, 2300 AA Leiden, The Netherlands; 2 Youz, Parnassia Group, The Hague, The Netherlands

**Keywords:** Treatment failure, Drop out, Children, Mental health care, Adolescent psychiatry, Systematic review

## Abstract

Youth with severe and enduring mental health problems (SEMHP) tend to drop out of treatment or insufficiently profit from treatment in child and adolescent psychiatry (CAP). Knowledge about factors related to treatment failure in this group is scarce. Therefore, the aim of this systematic review was to thematically explore factors associated with dropout and ineffective treatment among youth with SEMHP. After including 36 studies, a descriptive thematic analysis was conducted. Themes were divided into three main categories: client, treatment, and organizational factors. The strongest evidence was found for the association between treatment failure and the following subthemes: type of treatment, engagement, transparency and communication, goodness of fit and, perspective of practitioner. However, most other themes showed limited evidence and little research has been done on organizational factors. To prevent treatment failure, attention should be paid to a good match between youth and both the treatment and the practitioner. Practitioners need to be aware of their own perceptions of youth’s perspectives, and transparent communication with youth contributes to regaining their trust.

**Supplementary Information:**

The online version contains supplementary material available at 10.1007/s00787-023-02182-z.

## Introduction

There is a small group of youth, with a multitude of classifications, who suffer from severe and enduring mental health problems (SEMHP). These youth, aged 12–25 years, have interrelated and structural mental health problems that necessitate care, that lead to serious limitations in psychosocial functioning. This group often drops out or insufficiently profits from treatment in child and adolescent psychiatry (CAP) [1–3]. Youth with SEMHP often show persistent self-destructive behavior and frequently experience trauma, social exclusion, abuse, homelessness, problems with substance use, or involvement with the criminal justice system [4–6]. Recent studies have shown that the complexity of mental health problems for this group has increased over the last decade, with a marked increase in self-harming behavior [7]. In addition to the increasing complexity of mental health problems, there is also a societal concern due to rising waiting lists for specialized psychiatric treatment and high costs of healthcare [8, 9].

When severe mental health problems in adolescence remain untreated, they often lead to long-term mental health problems and dysfunction in adulthood [10]. Hence, treating youth with SEMHP as early and effectively as possible is of great importance. The need to improve mental health care for youth with SEMHP, more personalized treatment [11], and prevention of treatment failure is broadly recognized in the field [6, 12]. In addition, there has been extensive research on different influences of access to care and service use among youth [13, 14]. These studies have established valuable frameworks in which the dynamic nature of service use and core factors (e.g., contextual and mediating individual factors) affecting the use of care are described. However, we lack understanding of why current care does not meet the needs of this specific group of youth with SEMHP, who do not profit or drop out of care. Therefore, we need to gain knowledge on factors related to treatment failure, to improve care, and thereby better support the needs of youth with SEMHP.

Treatment failure can be defined as ineffective treatment and/or dropout, with both being interconnected. Ineffective treatments do not cause any or sufficient relief, leading to high dropout rates [15, 16]. Dropout is generally defined as a patient terminating treatment for mental health problems before the treatment provider assumes that treatment is complete [17]. A specific form of dropout is pushout: discontinuation of treatment initiated by the practitioner against the will of the adolescent [18]. As a result of dropout, a deterioration of mental health problems and therapy resistance can occur [6]. Therapy resistance is frequently associated with high perceived burdensomeness and suicidal behavior, which we often see in youth with SEMHP [6, 11, 19].

In current practice, clinicians regularly assign an evidence-based therapy to a patient based on a classification [20]. For youth with SEMHP, this is a problem which might lead to higher rates of treatment failure. Specifically, youth with SEMHP have multiple, heterogenic, and complex mental health problems that change over time and are difficult to capture in a single classification [21]. As a consequence, current treatment, aimed at ‘fixing’ a single DSM classification is often insufficient to cover the wide range of mental health problems that occur in youth with SEMHP [12]. Therefore, examining factors associated with treatment failure in youth with SEMHP, beyond a specific classification or treatment modality, is of major importance [6].

The aim of this systematic review is to increase our knowledge about factors associated with treatment failure in youth with SEMHP. Because this study focuses on a specific group of 'youth with SEMHP' that has been rarely studied before, we expect to find few studies that meet inclusion criteria and we, therefore, opt for an open, explorative approach. By conducting a systematic review, a descriptive overview of factors specified on three levels will be provided: (a) client level (i.e., client characteristics, such as diagnostic classification, demographics, psycho-social factors, and systemic functioning), (b) treatment level (i.e., characteristics involving type of treatment, turnover in therapists and therapeutic factors, such as alliance) and (c) organizational level (i.e., health care policy and treatment transcending characteristics, such as waiting lists and cost of healthcare). With this knowledge we can guide practice how to improve current treatment for youth with SEMHP, to better fit their needs and, thereby, limit treatment failure.

## Methods

A research protocol to guide this systematic review was prospectively registered in the International Database of Prospectively Registered Systematic Reviews in Health and Social Care (registration number CRD42021238294). The Preferred Reporting Items for Systematic Reviews and Meta-Analysis (PRISMA) guidelines were followed to guide and transparently report the findings from this review process [22].

### Search strategy

The search strategy was established in collaboration with a medical research librarian from the Leiden University Medical Centre. Search terms were related to the following areas of interest: (a) youth with severe and enduring mental health problems (SEMHP), such as child, pediatric, adolescents, and youth; (b) in combination with mental health problems, psychiatric disorders, severe and enduring including their synonyms; and (c) dropout and ineffective treatment, such as patient dropout, premature termination, treatment failure, and non-response. The full search strategy is provided in Appendix A. The following electronic databases were searched: PubMed, PsycINFO, MEDLINE, Cochrane Library, and Web of Science. Additional articles were selected by screening reference lists of included studies.

### Eligibility criteria

To be included, studies had to meet the following eligibility criteria:

- Focus on children and adolescents (youth) aged 12–25 years. Studies with a broader age range were included as long as the mean age of the participants fell between 12 and 25 years.
- Focus on youth in treatment at child and adolescent psychiatry services (CAP), due to severe and enduring mental health problems (SEMHP). To be included in this review, severe and enduring mental health problems were defined as: (1) interrelated and structural that (2) necessitate care and (3) lead to serious limitations in psychosocial and systemic functioning. Our description of SEMHP was derived from the definition of severe and enduring psychiatric disorders for a broader population [23].
- Type of setting: child and adolescent psychiatry (CAP), i.e., treatment facilities focusing on recovery or a reduction of psychiatric mental health problems. Treatments may include psychotherapy, behavioral therapies, and admission to a day-treatment or (semi-) residential treatment. Preventative care and care not focused on recovery or improvement of mental health problems, for example, preventative mental health programs at schools, were no part of this study.
- Outcomes with a focus on: (1) treatment outcomes (e.g., partial remission or exacerbation of mental health problems); (2) factors explaining dropout or ineffective treatment (e.g., pre-treatment client characteristics, treatment, and/or organizational characteristics); and (3) factors in treatment influencing dropout or ineffective treatment (e.g., therapeutic alliance and/or engagement processes).
- Articles published between 1994 and May 2022 in peer-reviewed, English language journals. Rationale for the cutoff for 1994 is the publication of the fourth version of the Diagnostic and Statistical Manual of Mental Disorders (DSM) in 1994. Full-text had to be available.
- Study design: all study designs were included (qualitative, quantitative, and mixed-method), as we aimed to increase the likelihood of finding different factors: client, treatment, and organizational factors.

### Data extraction and syntheses

**Fig. 1:**
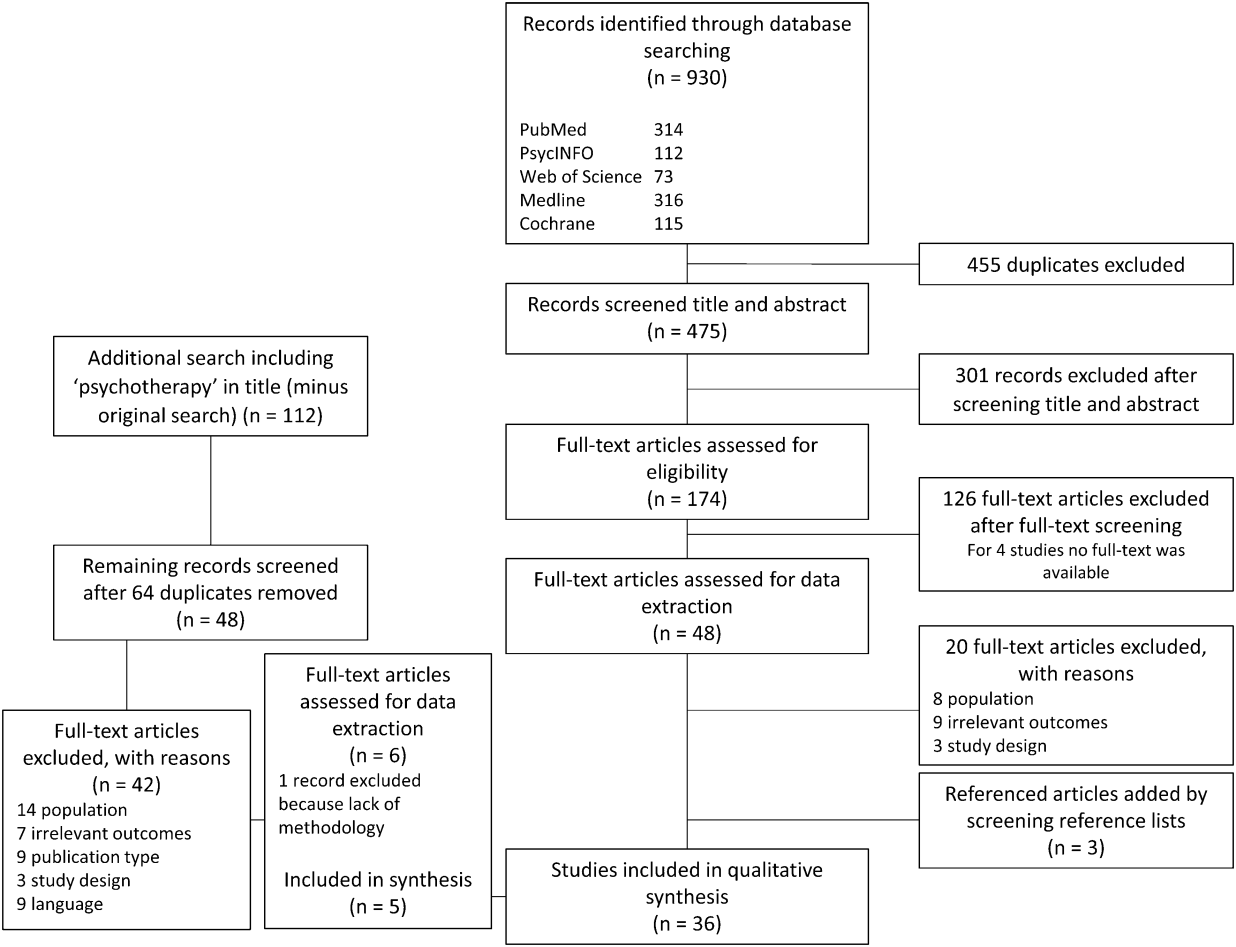
PRISMA flow chart

Study selection was carried out using Rayyan software [24]. Two reviewers (RS and CB) independently screened all titles and abstracts for eligibility using a PRISMA flow chart (see Fig. 1). Uncertainties were resolved by a third reviewer (HvE). After full-text screening, two reviewers (RS and CB) extracted and critically assessed the eligible studies using an a priori developed data extraction form. General information (e.g., study characteristics, definition of dropout, ineffective treatment results, target group and treatment) was registered on the extraction form and studies were again screened for eligibility. Any discrepancies between the two reviewers about the eligibility of studies were identified and resolved through discussion with a third reviewer (HvE or LAN).

To synthesize the data, a thematic data analysis was executed. This approach consisted of three steps, starting with open coding: line-by-line coding of the results by the reviewers (RS and CB) for each included article. Through discussion with the research team, this approach ensured agreement as to whether the extracted themes answered our research questions. Open coding was followed by axial coding: descriptive themes were developed by grouping together similar codes from the open coding phase, and by creating an overarching code to cover these first codes [25]. For each theme, all evidence was listed for factors associated with dropout or ineffective treatment. Finally, to go beyond the descriptive themes, analytical themes were generated and divided into three categories (i.e., client factors, treatment factors, and organizational factors) to answer our review questions [26]. To prevent interpretation bias, a second reviewer (LAN) assessed the themes on relevance. All studies were controlled for repeated sample use to avoid publication bias. In eight studies, there was an overlap in using the same database as another study. This was taken into account in the weighting of the results.

### Quality appraisal

Quality of individual studies was assessed using critical appraisal checklists by the Joanna Briggs Institute, including checklists for qualitative studies, randomized controlled trials, cohort studies, case-control studies, and cross-sectional studies [27]. A ranking system was defined by the authors to objectively assess the studies’ methodology and possible bias in design. The ranking system was predefined as follows: high quality (more than 8 items checked), medium quality (6–8 items checked), and low quality (less than 6 items checked). All evidence was organized per theme and labeled based on the ranking system (high, medium, or low).

The strength of evidence was assessed for each subtheme by a grading system [28]. For an extensive description of the method, refer to Nooteboom et al. [29]. The criteria used to assess the strength of evidence can be found in Online Appendix B. Finally, the strength of evidence was determined for all themes, based on the scores on each criteria using the following categories: very strong (+++++), strong (++++), medium (+++), limited (++), or no evidence (±).

## Results

### Study selection

Our search resulted in 930 studies of which 475 were screened for title and abstract after removing duplicates (Fig. 1, PRISMA flow chart). After full-text screening, 48 articles were assessed for data extraction of which 20 articles were excluded. By screening reference lists, three referenced articles were included in the final qualitative synthesis. During the reference search, we concluded that the search term ‘psychotherapy’ did not appear as a title word in our original search strategy. It was, therefore, decided to carry out an additional search which resulted in 48 extra non-duplicate articles, of which five were included in the final data synthesis. In total, 36 articles were included in the qualitative synthesis.

### Study characteristics

An overview of the study characteristics can be found in Online Appendix C. The 36 included studies comprised a wide range of mental health problems: eating disorders (*n* = 7), personality disorders (*n* = 5), mood disorders (*n* = 4), anxiety disorders (*n* = 4), trauma-related disorders (*n* = 4), substance use disorder (*n* = 1) and various (*n* = 11). Treatment settings varied widely from inpatient treatment to outpatient programs, involving cognitive behavioral therapy, family-based therapy, individual psychotherapy, or group mentalized-based therapy. The majority of the study designs were descriptive (*n* = 27), followed by cohort studies (*n* = 4), RCTs (*n* = 3), cross-sectional (*n* = 1), and mixed-methods studies (*n* = 1). Critical appraisal resulted in 3 high quality studies, 23 medium quality studies, and 10 low quality studies.

### Outcomes

**Fig. 2:**
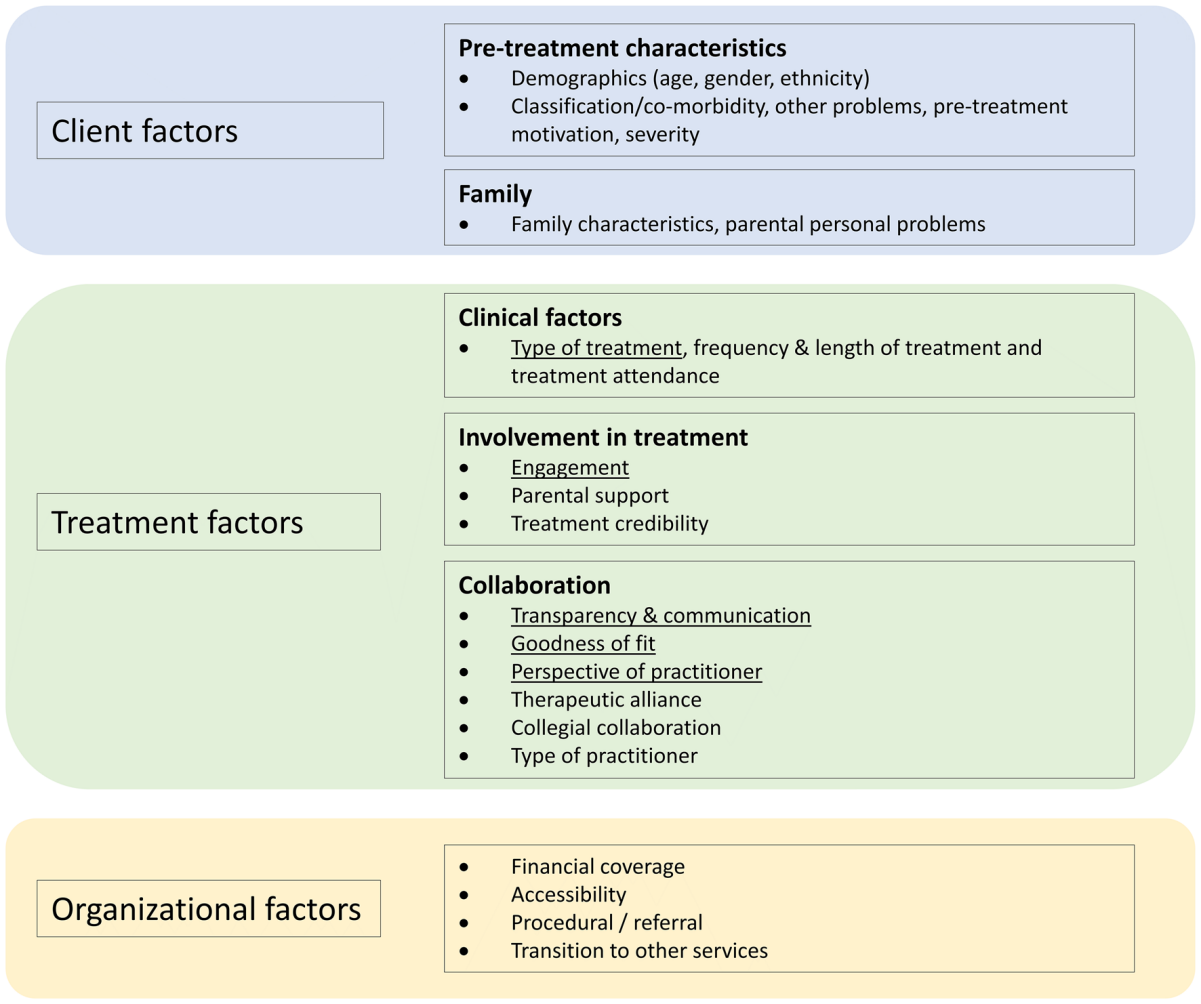
Descriptive overview of factors related to treatment failure in youth with SEMHP

Factors associated with treatment failure were divided into three categories: client factors, treatment factors, and organizational factors (Table 1). Each category consisted of main themes and subthemes, for which the strength of evidence was calculated (see Table 2 for the summary of findings). Figure 2 shows the results of the thematic analysis: a descriptive overview of factors related to treatment failure in youth with SEMHP. Overall, the strength of evidence for the subthemes was limited to medium. Regarding client factors, limited evidence was due to inconsistencies, meaning that one or more studies directly refuted or contested the findings of other studies carried out in the same context or under the same conditions. For organizational factors, the limited evidence was due to the low number of studies on this topic. However, for the subthemes ‘type of treatment’, ‘engagement’, ‘communication and transparency’ and, ‘goodness of fit’, the strength of evidence was medium to strong. The strength of evidence was strongest for the subtheme ‘perspective of practitioner’. The section below describes the findings of the thematic analysis in more detail.

**Table 1:** Study numbers per category

Category	Study numbers	Total
Client factors	30, 31, **32**, 33, 34, 35, 36, 37, 38, 39, 40, 41, 42, 43, 44, 45, 46, **47**, 48, 49, 50, 51, 52, 53, 54, **55**, 56, 57, 58, 59	28
Treatment factors	30, 31, **32**, 33, 34, 39, 42, 43, 44, 45, 46, **47**, 50, 51, 52, 53, 54, **55**, 56, 58, 59, 60, 61, 62, 63, 64, 65	25
Organizational factors	35, 37, 41, 45, 46, 51, **55**, 56	8

Weighting of related studies: A. study #28 and #38 count as one; B. study #42 and #54 count as one; C. study #51 and #63 count as one

Studies in high quality are indicated in bold

**Table 2:** Summary of findings table

Theme	Subtheme (number of studies) Study numbers	Quality (of individual studies)	Context	Consistency	Perspective	Strength of overall evidence
*Client factors*						
Pre-treatment client characteristics	Demographic—ethnicity (*n* = 6) 30, 31, 32, 33, 34, 35	High quality: 1 Medium quality: 4 Low quality: 1	Global	Inconsistent	Mixed	Limited–Medium
	Demographic— gender (*n* = 7) 32, 35, 36, 37, 38, 39, 40	High quality: 1 Medium quality: 3 Low quality: 3	Global	Inconsistent	Mixed	Limited–Medium
	Demographic—Age (*n* = 20) 30, 31, 32, 33, 34, 35, 36, 37, 38, 39, 40, 41, 42, 43, 44, 45, 46, 47, 48, 49	High quality: 2 Medium quality: 14 Low quality: 5	Global	Inconsistent	Mixed	Medium
	Classification/co-morbidity (*n* = 16) 30, 32, 35, 36, 37, 38, 39, 40, 44, 45, 47, 48, 50, 51, 52, 53	High quality: 2 Medium quality: 9 Low quality: 5	Global	Inconsistent	Mixed	Medium
	Other problems (*n* = 13) 31, 32, 33, 35, 36, 37, 39, 43, 46, 47, 49, 51, 54	High quality: 2 Medium quality: 9 Low quality: 2	Global	Mixed	Mixed	Medium
	Pre-treatment motivation (*n* = 7) 34, 39, 50, 51, 53, 55, 56	High quality: 1 Medium quality: 3 Low quality: 3	Global	Inconsistent	Mixed	Limited–Medium
	Severity (*n* = 17^1^) 30*, 32, 34, 35, 36, 37, 38, 39, 40*, 42, 45, 47, 48, 49, 50, 57, 58, 59	High quality: 2 Medium quality: 9 Low quality: 7	Global	Inconsistent	Mixed	Medium
Family	Family characteristics (*n* = 12) 30, 32, 33, 34, 35, 36, 37, 42, 43, 44, 46, 52	High quality: 1 Medium quality:10 Low quality: 2	Global	Inconsistent	Mixed	Limited–Medium
	Parental personal problems (*n* = 4) 33, 51, 53, 56	High quality: 0 Medium quality: 3 Low quality: 1	Global	Mixed	Mixed	Limited–Medium
*Treatment factors*						
Clinical factors	Type of treatment (*n* = 15) 30, 31, 32, 33, 39, 43, 44, 47, 50, 51, 52, 53, 54, 55, 60	High quality: 3 Medium quality: 9 Low quality: 3	Global	Mixed	Mixed	Medium–Strong
	Frequency and length of treatment (*n* = 5) 39, 42, 44, 55, 61	High quality: 0 Medium quality: 5 Low quality: 0	Global	Mixed	Mixed	Medium
	Treatment attendance (*n* = 5) 31, 32, 43, 44, 54	High quality: 1 Medium quality: 3 Low quality: 1	Global	Inconsistent	Mixed	Limited–Medium
Involvement in treatment	Treatment credibility (*n* = 7) 51, 53, 54, 55, 60, 62, 63	High quality: 1 Medium quality: 5 Low quality: 1	Global	Inconsistent	Mixed	Limited–Medium
	Engagement (*n* = 9) 34, 47, 50, 51, 54, 55, 60, 62, 63	High quality: 2 Medium quality: 4 Low quality: 3	Global	Consistent	Mixed	Medium–Strong
	Parental support (*n *= 8) 30, 34, 51, 53, 55, 56, 62, 63	High quality: 1 Medium quality: 5 Low quality: 2	Global	Mixed	Mixed	Medium
Collaboration	Therapeutic alliance (*n* = 11) 30, 32, 34, 54, 55, 56, 58, 61, 63, 64, 65	High quality: 2 Medium quality: 5 Low quality: 4	Global	Inconsistent	Mixed	Limited–Medium
	Collegial collaboration (*n* = 2) 51, 59	High quality: 0 Medium quality: 1 Low quality: 1	Global	Consistent	Mixed	Medium
	Transparency and communication (*n* = 5) 51, 55, 56, 60, 63	High quality: 1 Medium quality: 3 Low quality: 1	Global	Consistent	Mixed	Medium–Strong
	Goodness of fit (*n* = 4) 51, 55, 56, 63	High quality: 1 Medium quality: 2 Low quality: 1	Global	Consistent	Mixed	Medium–Strong
	Perspective of practitioner (*n* = 6) 47, 51, 54, 55, 56, 63	High quality: 2 Medium quality: 2 Low quality: 2	Global	Consistent	Mixed	Strong
	Type of practitioner (*n* = 3) 43, 45, 46	High quality: 0 Medium quality: 2 Low quality: 1	Global	Inconsistent	Single	No evidence
*Organizational factors*						
	Financial coverage (*n* = 2) 45, 56	High quality: 0 Medium quality: 0 Low quality: 2	Global	Mixed	Mixed	Limited–Medium
	Accessibility (*n* = 2) 51, 56	High quality: 0 Medium quality: 1 Low quality: 1	Global	Consistent	Mixed	Medium
	Procedural/referral (*n* = 6) 35, 37, 46, 51, 55, 56	High quality: 1 Medium quality: 4 Low quality: 1	Global	Inconsistent	Mixed	Limited–Medium
	Transition to other services (*n* = 2) 41, 51	High quality: 0 Medium quality: 2 Low quality: 0	Global	Consistent	Mixed	Medium

aWeighting of related studies: A. study #30 and #40 count as one, B. study #32 and #47 count as different studies, C. study #44 and #58 count as one, D. study #53 and #65 count as one

## Category 1. Client factors

The category ‘client factors’ is divided into two main themes: pre-treatment client characteristics and family characteristics.

### Main theme: pre-treatment client characteristics

This main theme includes demographics (i.e., subthemes: ethnicity, gender, age), factors associated with the diagnostic classification and co-morbidity, ‘other problems’, pre-treatment motivation, and factors related to the severity of problems. Limited to medium evidence was found for these subthemes, mainly due to inconsistencies in results of different studies.

#### Demographics

The three subthemes ethnicity, gender, and age did not appear to have a clear association with treatment failure. We found that the influence of ethnicity on treatment failure was inconsistent, with two studies pointing to an increased risk for ethnic minority youth to drop out of treatment [30, 31], and four studies reporting no evidence for any association between ethnicity and treatment failure [32–35].

Regarding the subtheme gender, most studies showed no association between gender and treatment failure [32, 35–39]. However, there was one study that found girls to be more likely to be non-responders in treatment [40].

The subtheme age during treatment was found in various studies as a significant predictor of dropout, with older adolescents being more likely to drop out [30–32, 37, 41–45]. However, other studies found youth who drop out of treatment to be younger [35, 46]. Moreover, in most studies, no evidence for any association between treatment failure and age was found [31, 33, 34, 36, 38–40, 47–49]. In addition, two studies reported that the age of onset was not related to treatment failure [38, 42].

#### Classification/comorbidity

Some studies found that having a co-morbid psychiatric disorder predicted greater dropout and lower rates of remission [44, 47, 50]. Different types of classifications were reported to be related to treatment failure, including conduct disorder [35], social phobia [50], borderline personality traits [51], substance use disorders [37, 45, 52], and co-occurring behavioral disorders [38, 45, 52]. Findings from one study suggested that compared with the other patient groups, mood disorders, especially major depression, were less common among dropouts [37]. On the contrary, there were also various studies that showed no evidence for a relationship between treatment failure and youth’s classification or comorbidity [30, 32, 36, 39, 40, 48, 53].

#### Other problems

Subtheme ‘other problems’ included various types of problems related to school, relationships, or with the law. We found consistent evidence in studies reporting that youth who dropped out showed a lack of stability in their life [35, 46, 47], more externalizing behavior, substance use, and problems with the law [32, 33, 36, 37, 47]. In addition, a few studies that included youth with various types of classifications reported that dropouts have lower internalizing and higher delinquent and externalizing behavior [31, 35, 39, 46]. However, one study on youth with Borderline Personality Disorder did not find that association [49]. Moreover, inconsistent results were found for the association between treatment failure and intellectual functioning [32, 36, 43, 49], self-harming behavior [37, 47] and, problems with developmental issues, such as sexuality [35, 46, 49, 51, 54].

#### Pre-treatment motivation

Youth’s preconceptions of mental health care and not being open to help were related to treatment failure according to some studies [50, 51, 55]. In addition, their own beliefs about their mental illness and fear of coping with their symptoms prevented youth from being motivated to change [50, 56]. However, other studies refuted this finding and found no evidence for the association between pre-treatment motivation and dropout [34, 39, 53].

#### Severity

Some studies showed that more severe symptoms (e.g., baseline severity, suicide attempt, hospital admittance, duration of illness) led to increased odds of dropout [30, 37, 38, 40, 42, 45, 48, 57], while studies also found contrary evidence for severity symptoms in association with treatment failure, such as prior treatment and duration of illness [30, 42, 48]. Other studies found no evidence for this association concerning the severity of illness in relation to treatment failure [32, 34–36, 39, 47, 49, 50, 58, 59].

### Main theme: family

The main theme ‘family’ consists of the subthemes ‘family characteristics’ and ‘parental personal problems’. Strength of evidence of these subthemes was limited to medium.

#### Family characteristics

Some studies found an association between dropout and SES [37], marital status [46], youth living with one parent [42], foster care [34, 37], and dysfunctional families [44, 52]. However, other studies refuted these findings and reported that treatment failure was not related to the number of parents [43], family caregiver structure [30, 35, 52], parenting styles [32], and SES [30, 33, 34, 36].

#### Parental personal problems

Some studies associated the following factors with dropout: psychological characteristics of parents (e.g., internalizing problems, difficulties in empathizing, and modulation of autonomy/dependency) and other responsibilities or conflict over care [51, 53, 56]. However, one study found that more engaged youth reported more family conflicts, thus being more motivated to change [33].

## Category 2. Treatment factors

### Main theme: clinical factors

The main theme ‘clinical factors’ comprises the type, frequency and length of treatment, and treatment attendance. Although the strength of evidence was limited for most of the subthemes, the strength of evidence for the subtheme ‘type of treatment’ was medium to strong.

#### Type of treatment

Studies reported that the type of treatment [31, 47, 52] and a lack of flexibility within the treatment format [51, 55] were related to dropout. Especially in group treatment, studies showed that youth had difficulties with the group format, because it felt unsafe to open up [50, 60]. Although, there were also studies that found no evidence for the association between the type of treatment and treatment failure, deeper analysis showed that most of these studies involved individual treatment [30, 32, 33, 39, 43, 44, 53, 54].

#### Frequency and length of treatment

Evidence regarding the frequency and length of treatment was mixed. On one hand, for youth with eating disorders, being hospitalized longer and more frequently was a risk for dropout [42, 44, 57]. On the other hand, studies of various other mental health problems reported that the treatment duration of dropouts was lower compared to completers [61], and treatment effectivity increased when treatment was more frequently provided [39].

#### Treatment attendance

Some studies showed that those with a history of missed appointments were more likely to drop out [31, 32, 43, 54]. However, another study found no relation between treatment attendance and ineffective treatment [44].

### Main theme: involvement in treatment

This main theme includes treatment credibility, engagement in treatment, and parental support. The strength of evidence for the subtheme ‘engagement’ was medium to strong, while the other subthemes were rated limited to medium.

#### Treatment credibility

Treatment credibility can be defined as the expectancies of youth or parents about how likely they are to benefit from a treatment and the diagnostic agreement. Some studies showed that disagreement on diagnosis and lower treatment credibility were associated with dropout, especially in children compared to adolescents [51, 53, 60, 62, 63]. Reported barriers were a narrow focus on assessment and classification [55]. However, another study refutes this finding, reporting that treatment expectancy was unrelated to dropout [54].

#### Engagement

Regarding the engagement of youth, studies reported that youth terminate treatment when they do not perceive it to be helpful [47, 54, 60, 62, 63]. Other factors associated with dropout were: being referred by adults instead of being self-referred [55], feeling unwanted [55], negative experiences with treatment [51], and having other priorities [51, 60]. In addition, the vicissitudes of treatment (e.g., annoyances associated with the constraints of treatment such as attending regularly, missing leisure activities, and talking about painful memories) could lead to avoidance and eventually dropout [34, 50, 60, 63].

#### Parental support

Various studies reported on the influence of parental support on treatment failure. Barriers experienced by parents (e.g., practical barriers, previous negative experiences, fear of stigma, and not seeing the need for treatment) were negatively related to treatment outcome [51, 62, 63]. Better caregiver participation, youth perceived parental approval, and less parental avoidance were associated with a lower risk of dropout [30, 34, 51, 53, 56, 63]. However, obliged involvement of parents could also lead to dropout [55]. Looking into the dynamics between caregiver participation and the child’s age in relation to treatment failure, one study found that the participation of caregivers in treatment was higher for younger youth [30].

### Main theme: collaboration

This main theme involves six subthemes: therapeutic alliance, collegial collaboration (including support and supervision), transparency and communication (i.e., the way practitioners communicate clearly and transparent with youth and their family), goodness of fit, the perspective of practitioner, and the type of practitioner. The strength of evidence was strongest for the subthemes transparency and communication, goodness of fit, and the perspective of practitioner.

#### Therapeutic alliance

Evidence indicates that a poorer therapeutic alliance with youth and parents increased the risk of treatment failure [30, 32, 34, 56, 58, 61, 64]. Reported barriers in the alliance were disinterest and insensitivity of the practitioner [54, 55], practitioners being too dominant in structuring the session [65], too much relational distance of the practitioner, and negative perceptions regarding the practitioner’s competence, personality, or motivations [63]. However, other studies found no relation between therapeutic alliance and treatment failure [34, 54, 64, 65]. In particular, alliance early in treatment appeared not to be related to treatment failure [58, 61]. Another study found that in the beginning of treatment, youth seemed to be more orientated towards their caregivers’ opinions, rather than their own relationship with their practitioner [30].

#### Collegial collaboration

Two studies reported on the relation between collegial collaboration and dropout. Results show less collegial alliance in practitioners treating patients who dropped out [59]. A lack of supervision and support was a treatment vulnerability related to premature termination [51].

#### Transparency and communication

Regarding transparency and communication, studies described that feeling pressured by the practitioner, communication issues such as annoyance with automated questions in treatment [55, 60, 63], and a lack of transparency and violations of trust [51, 55, 56] were related to treatment failure [55, 60, 63].

#### Goodness of fit

Goodness of fit was defined as the match between practitioner and client and practitioners’ disposition to treat. Although the number of studies in this theme was small (*n* = 4) and of medium quality, there was consistent evidence that treatment failure was associated with a ‘mismatch’ between youth and the practitioner [51, 55, 56, 63]. Practitioners’ insecurity and inauthenticity, inattentiveness, too much focus on efficiency, and practitioners’ low-risk tolerance were factors related to treatment failure [51, 55, 63].

#### Perspective of practitioner

Various studies reported on the association between practitioners’ perspectives on the clinical picture and treatment process, and treatment failure. Barriers included a judgmental approach, a rigid and narrow focus on problems [54, 55], and negative expectations regarding youth’s prognosis [51]. Moreover, studies described a lack of practitioners’ awareness of youth’s dissatisfaction and most often attributed barriers in treatment to youth and their parents, rather than to their own handling [47, 56, 63].

#### Type of practitioner

Some studies showed practitioners of youth who dropped out had less experience [46] and were non-specialists, compared to the practitioners of continuers [45]. However, another study found the treatment team and case manager discipline was not predictive of dropout [43].

## Category 3. Organizational factors

This category comprises four themes: financial coverage (i.e., factors involving insurance and other funding for treatment), accessibility (i.e., issues concerning locations and reaching practitioners), procedural/referral (i.e., issues with procedures of mental health care, referrals, workload, and scheduling), and transition to other services (i.e., transition from one specialized mental health institution to another or to adult care). Overall, evidence for the themes in this category was limited to medium in strength, mostly due to the small amount of studies in this topic.

### Financial coverage

One study found that financial access to services (youth covered by an insurance plan) did not guarantee sustained involvement in treatment [45]. Another study reported financial barriers associated with treatment failure were billing and insurance issues (e.g., funding for non-traditional activities) [56].

### Accessibility

Two studies reported that treatment failure was associated with the accessibility of services, including distance to service, issues with transportation, and technology issues related to accessibility, such as texting [51, 56].

### Procedural/referral

Treatment failure was related to a high therapist turnover and caseload [51, 55], scheduling issues [51, 56], unclear case management procedures and protocols for responding to risky behavior, and being forced to attend treatment [51, 55]. Two studies found participants who were referred by others (as opposed to being self-referred), were more likely to dropout [35, 46]. Another study showed no evidence for the effect of the kind of referral on treatment failure [37].

### Transition to other services

Readiness for transfer appeared to be related to treatment dropout [41]. The transition at age 18 was not beneficial for all youth and could lead to persistent mental illness and poor psychosocial outcomes [41, 51].

## Discussion

The aim of this systematic literature review was to thematically explore and describe factors associated with treatment failure among youth with severe and enduring mental health problems (SEMHP). To understand why youth with SEMHP drop out of treatment, the focus has long been on client factors, such as age, gender, and type of mental health problems. Interestingly, the results of this review emphasize that it is not the degree of severity or the diagnostic classification that determines treatment failure, but the context and living environment of youth that enables them to follow treatment, and how that treatment matches their needs. Therefore, to prevent treatment failure, we must pay attention to creating a stable environment on various life domains early in treatment. More importantly, we can conclude that focusing on treatment factors to prevent treatment failure of youth with SEMHP is crucial (i.e., type of treatment, engagement, transparency and communication, goodness of fit, and perspective of practitioner). The way practitioners interact and collaborate with youth is of utmost importance in the treatment to fit the needs of youth with SEMHP.

In this review, we found that youth’s treatment engagement is influenced by how treatment matches the needs of youth with SEMHP. In this regard, it is noteworthy that there is mixed evidence for the association of treatment failure and frequency and length of treatment in eating disorders compared to other diagnostic classifications. In youth with eating disorders, longer and more frequent hospitalization is associated with treatment failure. However, for other diagnostic classifications we did not see this association (subtheme ‘frequency and length of treatment’). This mixed evidence may indicate that treatment failure can be related to two different pathways. First, youth seem to drop out when treatment does not match their expectations, regardless of the length of treatment, when they feel unsafe because of, for example, the type of treatment (e.g., group treatment). Second, youth experience treatment failure when they lose hope after prolonged and multiple (crisis) admissions without any improvement. It is important to further study this hypothesis. An important characteristic of youth with SEMHP is that they often have negative experiences with previous treatments. As shown in other research, this can lead to a loss of hope if yet another treatment fails [6]. Consequently, for both pathways, treatment that does not fit youth’s needs can lead to iatrogenic harm, in which youth feel increasingly helpless, distrustful, and avoidant. We should be aware that helplessness of youth with SEMHP is not confused with a lack of motivation, and that it might take a long time before they feel safe in treatment, as shown by the study of Lundkvist-Houndoumadi and Thastum [50].

Interestingly, we found relatively stronger evidence for the relation between youth’s engagement and treatment failure, compared to the relation between treatment failure and pre-treatment motivation of youth and parents. Although a lack of engagement could be explained as a lack of (pre-treatment) motivation, as mentioned by Herpers et al. [6], evidence in this review (subtheme: ‘engagement’) shows that youth who were initially motivated for treatment ended up not being engaged, because they did not perceive the treatment to be helpful. Other research on service use similarly highlights the importance of youth's perceptions of the need and utility of treatment on their engagement [14]. To better support youth with SEMHP, practitioners should distinguish youth’s initial motivation for treatment and engagement during treatment, and take prior experiences with treatment (failure) into account. In this way, treatment failure will not be automatically assigned to youth or parents’ internal motivation, and solutions might be sought in a treatment that better fits their emotional and practical needs. Treatment that may relate to this is dialectical behavior therapy (DBT). DBT focuses on emotional dysregulation, particularly in youth with self-harm and suicidal behavior. DBT is preceded by a commitment phase, in which a commitment is made between youth and practitioner, strengthening the motivation for treatment and the therapeutic relationship [66, 67].

To improve treatment engagement of youth with SEMHP, evidence from the subtheme ‘parental support’ shows that caregivers’ participation could be of importance. Studies in our review underline that in early stages of treatment youth are often more oriented towards their caregivers’ opinion than to their own relationship with the practitioner. Moreover, we found that obliged caregiver involvement can lead to dropout. When caregivers express little confidence in treatment, it will affect the extent to which youth participate. Therefore, early involvement of caregivers in shared decision-making about, for example, the type of treatment, with youth’s consent, seems essential.

This review also underlines the importance of collaboration between youth and their practitioner. From the subthemes ‘transparency and communication’, ‘goodness of fit’ and ‘perspective of practitioner’, we can conclude that for youth with SEMHP an authentic therapeutic relationship is essential to regain trust in the practitioner and treatment. In this relationship, a practitioner should be able to communicate transparently. Moreover, in case of a mismatch between practitioner and youth, the risk of treatment failure is considerable (subtheme: ‘goodness of fit’). This is in line with previous research stating that to prevent dropout attention should be paid to the therapist–patient match and the quality of the therapeutic relationship [68]. However, long waiting lists, shortage of staff, and frequent changes of practitioners hinder practice from investing in this match [8]. This is an urgent bottleneck, which disadvantages youth with SEMHP and increases the risk of ineffective care, prolonged care trajectories, or treatment failure. Treatment approaches that provide time for a practitioner to get to know the youth and gain confidence, such as Youth Flexible Assertive Community Treatment, can be of added value in the treatment for youth with SEMHP [69].

Another finding of this review was the importance of the practitioners’ view on the diagnosis and treatment perspective of youth. As we know from previous research, youth with SEMHP can be challenging to treat, which can also negatively affect the practitioner [6]. Especially when youth lack trust and are suspicious, a negative interaction may arise between the youth and the practitioner, which puts considerable strain on the relationship and could lead to treatment failure. Practitioners might attribute barriers in treatment mostly to the youth and their parents, instead of examining their own part in the process (subtheme: ‘perspective of practitioner’). Therefore, we suggest that to improve the relationship between practitioners and youth, it is crucial that practitioners invest in self-reflection and frequent evaluations of the treatment process with youth and others involved, to make adjustments in time. Nickle et al. [70] shows that Feedback Informed Treatment has proven to be both a useful tool for these client-evaluations, as well as a way to explore and strengthen parental involvement, thereby reducing dropout.

Finally, we found small, but noteworthy, evidence on organizational factors in relation to treatment failure (themes: ‘accessibility’ and ‘transition to other services’). Specifically, we found studies that reported an association between treatment failure and the transition to adult care: the transition to adult mental health care at age 18 is arbitrary and not tailored to the ‘readiness’ of youth with SEMHP (theme: ‘transition to other services’). This, in combination with the importance of a stable living environment and increase of discontinuity of care during this vulnerable period, might put youth with SEMHP at a greater risk of treatment failure, as also reported in previous research [12]. Relevant research by Munson et al. [14] and Stiffman et al. [13] describe interacting factors that determine youth’s access to care and transition to adulthood. This shows that organizational factors play a major role on health care professionals’ behavior and subsequently youth’s care. Hence, more research, using a more dynamic framework, is needed on how to organize CAP in a way that youth with SEMHP are less likely to experience treatment failure due to the transition to adult care.

### Strengths and limitations

This study is unique for its comprehensive and exploratory view of youth with SEMHP, which entails strengths, but also limitations. First of all, the broad scope of this review allowed us to include many types of studies with a wide focus on various severe and enduring mental health problems in youth, together with a variety of treatments. Youth with SEMHP have never been studied as a group before due to its heterogeneity [71]. The broad inclusion criteria enabled us to examine what works for this group of youth, where classifications and treatment often overlap [72]. However, this broad approach also entails limitations. Overall, the subthemes explored in this review show a high degree of inconsistencies. This can be explained by the heterogeneous SEMHP group, where the mental health problems and the treatments offered vary. In addition, there is a risk of circular reasoning, since we study a group that is not yet well-defined. Although our description of youth with SEMHP is derived from the definition of Delespaul [23], how to interpret ‘severe’ and ‘enduring’ remains a point of debate. To minimize the risk of selection bias, we submitted a PROSPERO protocol prospectively that allowed us to work in a stepwise and transparent manner, following PRISMA guidelines.

Second, we have chosen an exploratory qualitative approach with a thematic analysis. We constructed themes out of data from different types of studies, giving meaning to treatment failure in this group of youth who often have different treatments and mental problems. Weighing the strength of evidence enabled us to give meaning to the importance of our findings. However, this thematic approach also has limitations that affect the way the results can be interpreted. It can be speculated that themes that we separated were actually interrelated, thereby increasing the risk of confirmation bias. We sought to minimize this risk through reflexive meetings to refute the formed themes and reach consensus on the final thematic model [73].

Third, this study identified factors associated with treatment failure without identifying the specific mechanisms by which they operate. For example, a lack of personal resources (financial coverage) could complicate accessibility to service. Difficulties with accessibility may in turn lead to a lack of perceived need and perceived efficacy of treatment, making youth vulnerable for treatment failure. Further research that explores the coherence between themes, taken into account the changes in youth moving through age and care systems, may provide in depth insight in these mechanisms. Finally, we included several pilot studies and studies with unclear inclusion criteria, leaving only a few high-quality studies for inclusion. To control for the quality of individual studies, we performed a quality assessment per (sub)theme. However, the inclusion of low-quality studies could have affected the strength of evidence of the individual themes and this should be taken into account when interpreting the results of this review. For future research, more high quality studies on this topic are needed.

## Conclusion

This review is the first to thematically explore factors related to treatment failure in youth with severe and enduring mental health problems in child and adolescent psychiatry. While the focus of explaining treatment failure has long been on specific client factors, this review suggests shifting the focus of practice and future research to treatment factors and the context of youth, to better support youth with SEMHP. Treatment should be tailored to the individual needs of youth and their caregivers to enhance engagement, considering previous experiences with treatment and the stability of the youth’s living environment. Moreover, practitioners should be aware of their own role in treatment, and the importance of transparent collaboration and a good fit. Finally, on an organizational level we should consider the way we currently organize mental health care services, with a cutoff at the age of 18 years, that does not seem compatible with the needs of youth with SEMHP.

## Supplementary Information

Below is the link to the electronic supplementary material.

Supplementary file1 (PDF 31 KB)

Supplementary file2 (PDF 35 KB)

Supplementary file3 (PDF 128 KB)

## Data Availability

The datasets generated and analysed during the current study are available from the corresponding author on reasonable request.
