# Supplementary material for: Drop-out and ineffective treatment in youth with severe and enduring mental health problems: a systematic review
Source: Eur Child Adolesc Psychiatry. 2023 Mar 7;33(10):3305–19. doi: 10.1007/s00787-023-02182-z (PMC11564352; doi:10.1007/s00787-023-02182-z)
Supplement: Supplementary file 1 — Supplementary file1 (PDF 31 KB) [file 787_2023_2182_MOESM1_ESM.pdf]

Appendix A. Search strategy

Pubmed search strategy on June 13 2022: 314 records

("child"[majr] OR "child"[ti] OR "children"[ti] OR "children's"[ti] OR "young adult"[majr] OR "adolescent"[majr] OR "Adolescent Behavior"[Majr] OR "schoolchild"[ti] OR "schoolchildren"[ti] OR "youngster"[ti] OR "youngsters"[ti] OR "boy"[ti] OR "boys"[ti] OR "girl"[ti] OR "girls"[ti] OR "adolescent"[ti] OR "adolescents"[ti] OR "adolescence"[ti] OR "schoolage"[ti] OR "schoolboy"[ti] OR "schoolboys"[ti] OR "schoolgirl"[ti] OR "schoolgirls"[ti] OR "prepuber"[ti] OR "prepubers"[ti] OR "prepuberty"[ti] OR "puber"[ti] OR "pubers"[ti] OR "puberty"[ti] OR "teenager"[ti] OR "teenagers"[ti] OR "teens"[ti] OR "youth"[ti] OR "youths"[ti] OR "underaged"[ti] OR "under-aged"[ti] OR "Pediatrics"[majr] OR "Pediatric"[ti] OR "Pediatrics"[ti] OR "Paediatric"[ti] OR "Paediatrics"[ti])

AND ("Mental Disorders"[majr] OR "Mental Disorder*"[ti] OR "mental health"[majr] OR "mental health problem*"[ti] OR "psychiatric disorder*"[ti] OR "mental health difficult*"[ti] OR "mental disease*"[ti] OR "mental illness*"[ti] OR "psychiatric disease*"[ti] OR "psychiatric illness*"[ti] OR "behavior disorder*"[ti] OR "behaviour disorder*"[ti] OR "behavioral disorder*"[ti] OR "behavioural disorder*"[ti] OR "behavior disorder*"[ti] OR "behaviour disorder*"[ti] OR "behavioral disorder*"[ti] OR "behavioural disorder*"[ti] OR "behavior problem*"[ti] OR "behaviour problem*"[ti] OR "behavioral problem*"[ti] OR "behavioural problem*"[ti] OR "internalizing"[ti] OR "internalising"[ti] OR "externalizing"[ti] OR "externalising"[tw] OR "anxiety disorder*"[ti] OR "PTSD"[ti] OR "posttraumatic stress disorder*"[ti] OR "post traumatic stress disorder*"[ti] OR "post-traumatic stress disorder*"[ti] OR "bipolar disorder*"[ti] OR "impulse control"[ti] OR "conductive disorder*"[ti] OR "disruptive disorder*"[ti] OR "oppositional defiant disorder*"[ti] OR ("autis*"[ti] AND "disorder*"[ti]) OR "ADHD"[ti] OR "Attention Deficit Disorder*"[ti] OR "mood disorder*"[ti] OR "depression*"[ti] OR "depressive disorder*"[ti] OR "schizophreni*"[ti] OR "psychos*"[ti] OR "psychotic"[ti] OR "personality disorder*"[ti] OR "substance related disorder*"[ti] OR "substance-related disorder*"[ti] OR "substance disorder*"[ti] OR "alcohol disorder*"[ti] OR "alcohol abus*"[ti] OR ("Marijuana"[ti] AND "abus*"[ti]) OR "Amphetamine-Related Disorder*"[ti] OR "Amphetamine Related Disorder*"[ti] OR ("XTC"[ti] AND "abus*"[ti]) OR "drug abus*"[ti] OR "drug use disorder*"[ti] OR ((("sever*"[ti] OR "intense"[ti] OR "suffering"[ti] OR "persisten*"[ti] OR "persisting"[ti] OR "enduring"[ti] OR "endures"[ti] OR "endure"[ti] OR "endured"[ti]) AND ("disorder*"[ti] OR "illness*"[ti] OR "morbidit*"[ti]) AND ("complex"[tw] OR "Comorbidity"[Mesh] OR "comorbid*"[tw] OR "multimorbid*"[tw])) OR (("sever*"[ti] OR "intense"[ti] OR "suffering"[ti] OR "persisten*"[ti] OR "persisting"[ti] OR "enduring"[ti] OR "endures"[ti] OR "endure"[ti] OR "endured"[ti]) AND ("disorder*"[tw] OR "illness*"[tw] OR "morbidit*"[tw]) AND ("complex"[ti] OR "Comorbidity"[Majr] OR "comorbid*"[ti] OR "multimorbid*"[ti]))))

AND ((("Patient Dropouts"[Mesh] OR "patient dropout*"[tw] OR "patient drop out*"[tw] OR "patients dropout*"[tw] OR "patients drop out*"[tw] OR "patient's dropout*"[tw] OR "patient's drop out*"[tw] OR "client dropout*"[tw] OR "client drop out*"[tw] OR "clients dropout*"[tw] OR "clients drop out*"[tw] OR "client's dropout*"[tw] OR "client's drop out*"[tw] OR "treatment dropout*"[tw] OR "treatment drop out*"[tw] OR "therapy dropout*"[tw] OR "therapy drop out*"[tw] OR ((patient*[ti] OR treatment*[ti] OR therap*[ti] OR CAP[ti]) AND (dropout*[ti] OR "drop out*"[ti])) OR "premature terminat*"[tw] OR (premature[ti] AND terminat*[ti]))) OR ((risk[tw] OR risks[tw] OR riskfactor*[tw] OR reason*[tw] OR barrier*[tw] OR challeng*[tw]) AND ("Treatment Failure"[Mesh] OR "treatment fail*"[tw] OR "therapy fail*"[tw] OR "ineffective treatment*"[tw] OR "ineffective therap*"[tw] OR "non-respons*"[tw] OR "nonresponse*"[tw] OR ((treatment*[ti] OR therap*[ti]) AND (fail*[ti] OR ineffect*[ti])))))
