# Supplementary material for: Drop-out and ineffective treatment in youth with severe and enduring mental health problems: a systematic review
Source: Eur Child Adolesc Psychiatry. 2023 Mar 7;33(10):3305–19. doi: 10.1007/s00787-023-02182-z (PMC11564352; doi:10.1007/s00787-023-02182-z)
Supplement: Supplementary file 2 — Supplementary file2 (PDF 35 KB) [file 787_2023_2182_MOESM2_ESM.pdf]

Appendix B. Strength of evidence

Criteria	Category	Indication	Description
Quality of individual study (based on individual study critical appraisal)	High	+	Over 75% of the studies was of high quality
	Medium	+/-	25-75 of the studies was of high quality
	Low	-	Under 25% was of high quality
Size of evidence[a]	Large	+	Over 15 individual studies
	Medium	+/-	Between 5 and 15 individual studies
	Small	-	Less than 5 individual studies
Context	Global	+	Findings came from different (therapeutic or type of mental health problem) contexts
	Specific	-	Findings came from a single therapeutic (e.g. residential care) context or outcome specific to a certain type of mental health problem (e.g. eating disorder)
Consistency	Consistent	+	All studies point to identical or similar conclusions
	Mixed	+/-	Studies based on a variety of different designs or methods applied in a range of contexts, have produced results that contrast with those of another study
	Inconsistent	-	One or more studies directly refutes or contest the findings of other studies carried out in the same context or under the same conditions
Perspective (source of evidence)	Mixed	+	Source of evidence comes from two or more participants: practitioners, parents and youth
	Single	-	Source of evidence from youth or practitioners or parents

[a] In case of two studies with the same dataset, this was counted as one study.
