# Supplementary material for: Drop-out and ineffective treatment in youth with severe and enduring mental health problems: a systematic review
Source: Eur Child Adolesc Psychiatry. 2023 Mar 7;33(10):3305–19. doi: 10.1007/s00787-023-02182-z (PMC11564352; doi:10.1007/s00787-023-02182-z)
Supplement: Supplementary file 3 — Supplementary file3 (PDF 128 KB) [file 787_2023_2182_MOESM3_ESM.pdf]

Appendix C. Study characteristics

Study number	Study (year)	Design (method)	Principal problem/ classification	Treatment	Respondents (n)	Quality
[30]	Ormhaug, and Jensen (2018)	RCT (Questionnaires)	PTSD	Individual trauma-focused CBT or TAU	Youth, parents and practitioners (n=156)	Medium
[31]	Fraynt, Ross, Baker, Rystad, Lee, and Briggs (2014)	Cohort study (retrospective)	Trauma	Outpatient care	Youth (n=562)	Medium
[32]	O'Keeffe, Martin, Goodyer, Wilkinson, Consortium, and Midgley (2018)	RCT (Interviews, questionnaires)	Depression	Individual BPI, CGT and STPP	Youth and parents (n=453)	High
[33]	Dakof, Tejeda, and Liddle (2001)	Descriptive (Interviews, questionnaires)	Drug abuse	MFT and individual CBT	Youth and parents (n=224)	Medium
[34]	Yasinski, Hayes, Alpert, McCauley, Ready, Webb, and Deblinger (2018)	Descriptive (Questionnaires, audio-observations)	Trauma	TF-CBT	Youth, caregivers and practitioners (n=108)	Low
[35]	Baruch, Gerber, and Fearon (1998)	Descriptive (Clinical data)	Various	Psychotherapy	Youth (n=134)	Medium
[36]	Gilbert, Fine, and Haley (1994)	Descriptive (Interviews, questionnaires)	Mood disorder	Group therapy	Youth (n=65)	Low
[37]	Pelkonen, Marttunen, Laippala, and Lönnqvist (2000)	Descriptive (Interviews)	Various	Outpatient care	Youth (n=297)	Medium
[38]	Masi et al. (2012)	Descriptive (File-analysis)	Social anxiety disorder	Psychotherapy and medication	Youth (N=140)	Low
[39]	Gergov, Lindberg, Lahti, Lipsanen, and Marttunen (2021)	Descriptive (Questionnaires)	Various	Psychotherapy	Youth (n=58)	Medium
[40]	Lindebø Knutsen, Sachser, Holt,	Descriptive (Interviews, questionnaires)	PTSD	TF-CBT	Youth (n=155)	Low

	Goldbeck, and Jensen (2020)					
[41]	Memarzia, St Clair, Owens, Goodyer, and Dunn (2015)	Cohort study (Interviews, questionnaire)	Not applicable	Not applicable	Youth (n=53)	Medium
[42]	Hubert et al. (2013)	Descriptive (Questionnaires)	Anorexia Nervosa	Inpatient treatment	Youth (n=293)	Medium
[43]	Johnson, Mellor, and Brann (2009)	Descriptive (File-analysis)	Various	Unclear	Youth (n=520)	Medium
[44]	Lock, Couturier, Bryson, and Agras (2006)	Cohort study (Interviews, questionnaires)	Anorexia Nervosa	FBT	Youth and parents (n=86)	Medium
[45]	Harpaz-Rotem, Leslie, and Rosenheck (2004)	Descriptive (File-analysis)	Various	Outpatient care	Youth (n=11.659)	Low
[46]	Baruch, Vrouva, and Fearon (2009)	Descriptive (Questionnaires)	Various	Psychotherapy	Youth (n=882)	Medium
[47]	O'Keeffe, Martin, Target, and Midgley (2019)	Mixed methods (RCT/Interviews)	Depression	Individual BPI, CBT and STPP	Youth and practitioners (n=99, interviews n=32)	High
[48]	Delinsky, et al. (2010)	Descriptive (Interviews, questionnaires)	Eating disorder	Residential care	Youth (n=80)	Medium
[49]	Jørgensen, Bo, Vestergaard, Storebø, Sharp, and Simonsen (2021)	Cohort (Questionnaires)	Borderline Personality Disorder	Group-MBT	Youth (n=89)	Medium
[50]	Lundkvist-Houndoumadi, and Thastum (2017)	Descriptive (Interviews, questionnaires)	Anxiety disorder	Group CBT	Youth and parents (n=106)	Low
[51]	Desrosiers, Saint-Jean, and Breton (2015)	Descriptive (Interviews)	Borderline Personality Disorder	Psychotherapy, DBT and parental guidance	Youth, parents and practitioners (n=12 trajectories)	Medium
[52]	King, Hovey, Brand, Wilson, and Ghaziuddin (1997)	Descriptive (Questionnaires, telephone interviews)	Suicidality	Multimodal treatment: medication, individual therapy and family therapy	Youth and Parents (n=66)	Medium

[53]	Wergeland, et al. (2015)	RCT (Interviews, questionnaires)	Anxiety disorder	Individual and group CBT	Youth and parents (n=182)	Medium
[54]	Fredum, et al. (2021)	Cross-sectional (Interviews, questionnaires)	Major depressive disorder	Individual STPP	Youth and Practitioners (n=69)	Low
[55]	Stige, Barca, Lavik, and Moltu (2021)	Descriptive (Interviews)	Various	Individual therapy with involvement of family	Youth (n=12)	High
[56]	Gearing, Schwalbe, and Short (2012)	Descriptive (Focus groups)	Various	Eclectic	Practitioners (n=34)	Low
[57]	Hergenroeder, Wiemann, Henges, and Dave (2015)	Descriptive (Retrospective chart review)	Eating disorder	Inpatient and outpatient care	Youth (n=218)	Medium
[58]	Pereira, Lock, and Oggins (2006)	Descriptive (Interviews, audio-observations)	Anorexia Nervosa	FBT	Youth and parents (n=41)	Medium
[59]	Murray, Griffiths, and Le Grange (2014)	Descriptive (Questionnaires)	Anorexia Nervosa	FBT	Practitioners (n=27)	Low
[60]	Andersen, Poulsen, Fog-Petersen, Jørgensen, and Simonsen (2021)	Descriptive (Interviews)	Borderline Personality Disorder	Group-MBT	Youth (n=10)	Medium
[61]	Hauber, Boon, and Vermeiren (2020)	Descriptive (Questionnaires)	Personality disorder	Group-MBT	Youth (n=105)	Medium
[62]	Jensen-Doss, and Weisz (2008)	Descriptive (Interviews, questionnaires)	Various	Unclear	Parents (n=197)	Medium
[63]	Desrosiers, Saint-Jean, Laporte, and Lord (2020)	Descriptive (Interviews)	Borderline Personality disorder	Psychotherapy and parental guidance	Youth, parents and practitioners (n=11 trajectories)	Medium
[64]	Isserlin, and Couturier (2012)	Descriptive (Questionnaires, observations)	Anorexia Nervosa	FBT	Youth and parents (n=14)	Low
[65]	Fjermestad, et al. (2021)	Descriptive (Questionnaires, observations)	Anxiety disorder	individual CBT	Youth and Practitioners (n=73)	Medium
